# Embryological Basis and Genetic Considerations of Right Lumbar Undescended Caecum with Paracaecal Appendix and Aberrant Vascular Supply

**DOI:** 10.15388/Amed.2026.33.1.23

**Published:** 2026-07-22

**Authors:** Anand Verma, Madhumita Patnaik, Praveen Kumar Ravi, Sayan Biswas, Manisha R. Gaikwad, Aarthi Manokaran

**Affiliations:** Department of Anatomy, All India Institute of Medical Sciences Bhubaneswar, India E-mail:

**Keywords:** genetic factors, malrotation, paracaecal appendix, undescended caecum, genetiniai veiksniai, malrotacija, paracekalinis apendiksas, nenusileidusi akloji žarna

## Abstract

The caecum normally descends to the right iliac fossa during embryogenesis. Disruptions in midgut development may result in its atypical positioning. In a male cadaver, the small, conical caecum and paracaecal appendix were in the right lumbar region. The ileocaecal orifice was on the anterior caecal wall, and the appendicular orifice opened on the medial caecal wall inferior to it. The ascending colon was short, and the hepatic flexure was inferior to hepatic quadrate lobe. The right colic artery was absent. The ascending branch of the ileocolic artery supplied the caecum, and the appendix. The disposition of the rest of the intestinal tract and peritoneum were observed, and measurements were taken. Midgut development involves multiple regulatory genes, signalling pathways, transcription factors and mesenchyme-endoderm cross-talk. Mutations in several candidate genes were identified as contributing to midgut malrotation. Awareness of such a rare anatomical variation is essential to avoid misdiagnosis of atypical appendicitis or intestinal obstruction.

## Abstract (it)

Įprastai embriono genezės metu akloji žarna nusileidžia į dešiniąją klubinę duobę. Vidurinės žarnos vystymosi sutrikimai gali lemti jos netipinę padėtį. Vieno vyro lavone maža, kūgio formos akloji žarna ir prieaklinis apendiksas buvo dešinėje juosmens srityje. Ileocekalinė anga buvo aklosios žarnos priekinėje sienoje, o apendikulinė anga atsivėrė vidinėje aklosios žarnos sienoje žemiau jos. Kylanti gaubtinė žarna buvo trumpa, o kepenų linkis buvo žemiau kepenų kvadratinio skilvelio. Dešinės gaubtinės žarnos arterijos nebuvo. Kylanti arterijos šaka aprūpino krauju akląją žarną ir apendiksą. Buvo matomas likusios žarnyno dalies ir pilvaplėvės išsidėstymas bei atlikti matavimai. Vidurinės žarnos vystymąsi lemia daug genų, signalinių kelių, transkripcijos veiksnių ir mezenchimos bei endodermos sąveika. Nustatyta, kad kelių kandidatinių genų mutacijos prisideda prie vidurinės žarnos malrotacijos. Žinoti apie tokią retą anatominę variaciją yra būtina, siekiant išvengti klaidingos netipinio apendicito ar žarnyno obstrukcijos diagnozės.

## Introduction

The caecum and appendix are usually located in the right iliac fossa [1,2]. The caecal bud appears during the sixth week of gestation from the post arterial segment of the midgut loop [3]. Midgut development is a complex process of physiological herniation, rotation, retraction of loops, differential growth and fixation. Any irregularities in these processes may lead to a malrotated midgut [1,3]. Autopsy studies indicate that intestinal rotational disorders occur in about 1% of the total population [4,5]. As a matter of fact, 95% of cases are diagnosed in infancy, and midgut malrotation getting diagnosed in adulthood is uncommon, having an incidence between 0.0001% to 0.19%, and showing a slight female predominance [5]. Appendicitis in a lumbar caecum and appendix poses a significant challenge during diagnosis and surgery [6–9]. The malrotated midgut may be associated with variant blood supply, leading to complications during colonoscopy and surgery. We present a case of a right lumbar undescended caecum with associated vascular variation.

## Case Report

**Figure 1:**
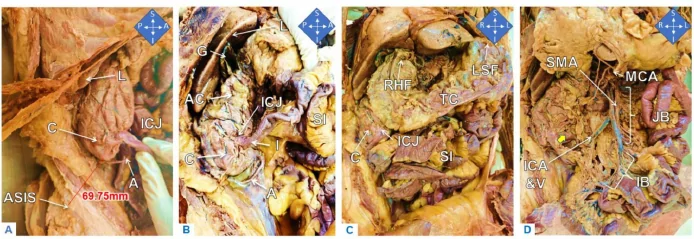
(A) Showing the position of the caecum in the lumbar region and the distance between the base of the caecum and the anterior superior iliac spine. (B) Lumbar caecum with the paracaecal appendix opening on its medial aspect, with the yellow arrow showing the pointed caecal apex. C) Right hepatic flexure positioned below the quadrate lobe of the liver. (D) Branching pattern of the superior mesenteric artery, with the yellow arrow showing the pointed caecal apex. *Note*. C – Caecum; ASIS – Anterior Superior Iliac Spine; AP – Appendix; RHF – Right Hepatic Flexure; ICA&V – Ileocolic Artery and Vein; ICJ – lleocaecal Junction; QL – pQuadrate Lobe; IL – Ileum; AC – Ascending Colon; SI – Small Intestine; G – Gallbladder; LI – liver; LSF – Left Splenic Flexure; SMA – Superior Mesenteric Artery; MCA – Middle Colic Artery; JB – Jejunal Branches; IB – Ilial Branches; A – Anterior; P – Posterior; S – Superior; I – Inferior; R – Right side; L – Left side.

During undergraduate cadaveric dissection on a 68-year-old donated male cadaver, we discovered that the caecum and appendix were located in the right lumbar region. The distance from the base of the caecum to the anterior superior iliac spine was 6.97cm (Fig. 1A). The caecum was intraperitoneal, 3.77cm long and 3.80 cm wide at the ileocaecal junction. The caecal base was directed medially, with the ileocaecal orifice facing anteriorly, and the appendicular orifice opened on the medial caecal wall inferior to the ileocacecal orifice. The caecum had a large right sacculation and was conical in shape, with the appendix attached to its apex. The appendix was 7.22 cm long, paracaecal in position, and suspended by the mesoappendix (Fig. 1B). The ascending colon was 9.41 cm long and retroperitoneal in position. The right hepatic flexure was positioned below the hepatic quadrate lobe. The transverse colon descended for a short distance from the right hepatic flexure overlapping the anterior surface of the ascending colon and then continued horizontally to the left side with its concavity directed upwards (Fig. 1C). The rest of the intestinal tract was in normal position. Ladd’s bands were absent. The disposition of the peritoneum was normal.

The ileocaecal artery (ICA) arose from the *Superior Mesenteric Artery* (SMA) descended to the right side, and bifurcated into ascending and descending branches. The descending branch of ICA supplied the distal ileum and anastomosed with the final ileal branch of the terminal part of the SMA. The caecum, appendix, and the ileocolic junction were supplied by the ascending branch of the ICA. The ascending colon was supplied by the anastomosis between the ascending branch of ICA and the two descending branches from the right branch of the *Middle Colic Artery* (MCA). The right colic artery was absent (RCA) (Fig. 1D).

The morphometric analysis for the linear dimensions and distances between specific anatomical landmarks were done with a vernier caliper, and the lengths of curved or irregular anatomical structures were measured with a thread. To determine the diameter of circular structures, the circumference was measured by using a thread, and the diameter was then calculated by using the formula: Diameter = Circumference ÷ π

## Discussion

The atypical location and morphology of the caecum and appendix may cause a misdiagnosis and delayed treatment of acute appendicitis, caecal volvulus and intestinal obstruction as well as increased complications during surgery and colonoscopy [1,5,6,7,9,10]. An undescended caecum and appendix occur due to disruptions in the normal midgut development [1,9].

During the sixth week, the rapidly elongating midgut loop and its mesentery temporarily undergo physiological umbilical herniation. By the tenth week, the intestines re-enter the abdominal cavity due to the regression of the mesonephric kidney, a slowed liver growth, and abdominal expansion following 270º counter-clockwise rotation around the axis of the SMA [1,9]. The caecal bud is the last part of the gut tube to re-enter the abdominal cavity and is initially in the sub-hepatic position [1,3]. The differential growth of midgut derivatives, specifically, the elongation of the ascending colon, the shrinkage in the right hepatic lobe, and gravity, allow its descent to the right iliac fossa at the 11^th^ week [6,11,12].

Abnormalities in midgut rotation, fixation and/or peritoneal fusions during the fetal life can cause atypical caecal positions like pelvic, lumbar, or subhepatic [1,9,10,12–14]. In our case, the ascending colon was short (its length was 9.41 cm instead of the usual 15cm), hence, as the abdominal cavity enlarged, the caecum and the appendix failed to descend normally, and remained in the lumbar region [10,11,15]. An undescended caecum in the lumbar region, while rare, has been documented earlier [9,13,14]. Multiple regulatory genes, signalling pathways, transcription factors and mesenchyme-endoderm cross-talk are instrumental in midgut development [12]. Both canonical and non-canonical signalling systems like a wingless-related integration site (Wnt), bone morphogenetic protein (BMP), Notch, and Sonic hedgehog are involved in the development of the large intestine [11,12]. Isolated intestinal malrotation shows autosomal dominant inheritance, while syndromic intestinal malrotations usually have autosomal recessive inheritance. Intestinal malrotation is associated with the Limb-Body Wall Complex and Fryns syndrome [16]. BCL6 gene mutation causes malrotation with an abnormal position of caecum and the presence of Ladd’s bands [17]. The dorsal mesentery is pivotal in the left–right asymmetry needed for gut rotation. The genes of FOXF1, Irx3, Pitx2 and Isl1 are selectively expressed on the left side of the mesentery under the control of the signalling molecule Nodal. Inactivation of heterozygous mutations of FOXF1 causes intestinal malrotation and alveolar capillary dysplasia. In humans, the FOX transcription factor cluster is located at 16q24.1. Chromosomal abnormalities – like 16q24.1 deletion, 16qduplication, ring chromosome 4 and 13q deletions – have been implicated in intestinal malrotation [16].

In the early fetal life, the caecum is short and conical; then, the right caecal saccule grows faster than the left, carrying the appendix towards the ileocaecal junction [15]. The intrinsic growth factors regulating the differential growth of the caecal wall and gravity (weight-bearing of caecal contents) lead to transition from infantile to adult caecal morphology [3,6,13]. Here, the conical caecum was smaller than usual, with a medially directed base formed by the larger right saccule. The orifice of the paracaecal appendix, though inferior to the ileocaeacal junction, opened medially on the caecum instead of the usual posteromedial position. Delic et al. found the appendicular orifice in the medial wall of caecum in 32% of cases [18]. In this study, the caecum can be classified as modified type III of the Treve’s classification [13,10]. We found the ileocaecal orifice on the anterior caecal wall. Whereas, Vidya C. S. et al. have noted that the ileocaecal orifice opened anteromedially in 10.8% of the specimens studied [19]. To the best of our knowledge, such atypical placement of both the ileocaecal and appendicular orifices in the same case has not been reported previously.

Normally, the caecum and the appendix are supplied by the descending branch of ICA [9,15]. In this case, the caecum, the appendix, and the ileocaecal junction were supplied by the ascending branch of ICA. A similar finding has been reported earlier [9]. Here, the descending branch of the ICA supplied the distal ileum and anastomosed with the final ileal branch of the SMA. One study the descending branch of ICA was slender, did not give any branches, and ended by anastomosing with the SMA [9]. We saw that the RCA was absent, and the ascending colon was supplied by the anastomosis of the ascending branch of the ICA with the MCA. Existing literature shows that the RCA may be absent in <5% of cases [15]. In 10% cases, an accessory RCA arising from the ICA supplies a normally positioned or undescended caecum and appendix [8,13,15]. The findings of this case report and comparable previous cadaveric studies have been summarised in Table 1.

**Table 1:** Summary of anatomic variations in presented in this case report and in relevant existing cadaveric studies

Parameter	Our study	Balasubramanian et al. (2013)	Ashwiniet al. (2014)	Jacob et al. (2013)	Savithri et al. (2013)	Ravi et al. (2017)
**1. Caecum** a) Location:	Right lumbar	Right lumbar	Right lumbar	Right lumbar	Subhepatic	Subhepatic
b) Morphometry (Length ×Breadth)	3.77×3.8cm	5×5.25cm	Not commented	Not commented	5×6cm	4×3.5cm
c) Type (Treves classification):	Modified type III	Conical (type I)	Not commented	Not commented	Exaggerated type (type IV)	Conical (type I)
**2. Vermiform appendix** a) length	7.22 cm	Not mentioned	8cm	Not commented	11 cm	11.5 cm
b) type of	Paracaecal	Retrocaecal	Subcaecal, highly coiled and retroperitoneal	Not commented	Not commented	12’o clock position/retrocaecal
c) orifice position (in caecum)	medially located and inferior to the ileocecal orifice	posteromedial and 2.75cm to the ileocaecal junction	Not commented	Not commented	Posteromedial and 1 cm below ileocaecal opening	
d) mesoappendix	present	absent	Not commented	Not commented	Not mentioned	Not commented
**3. Ascending colon** Length and breadth	L=9.41cm	L=12.5cm B=10cm	Not commented	L=8cm	Not commented	L=4.5 cm completely intraperitoneal
**4. Opening of ileocecal orifice**	Anterior wall of caecum; antero-superior to appendiceal opening	Posteromedial	Posteromedial	Not commented	Posteromedial	Posteromedial
**5. Variation in other parts of intestine and peritoneum (including Ladd’s band)**	None	None	None	Descending colon closed to right side in front of the great vessels and continued as sigmoid colon in right iliac fossa. These were covered with mesentery	Sigmoid colon was right-sided	The ascending part of terminal ileum (20cm long) had a narrow external diameter; Root of mesentery attached the caecum and ascending colon to the posterior abdominal wall.
**6. Blood supply of caecum and appendix**	Ascending branch of ileo-colic artery, branch of superior mesenteric artery	Not commented	Ascending branch of ileo-colic artery, branch of superior mesenteric artery	Not commented	Not commented	Ileo-colic artery, branch of superior mesenteric artery
**7. Associated vascular variation**	The right colic artery was absent	Not commented	The descending branch of ileocolic artery was very slender and did not give off any branch, and ended by anastomosing with the superior mesenteric artery.	The inferior mesenteric artery arose from the right side of the abdominal aorta, descended retroperitoneally and gave off sigmoid branches to the right side.	None	Accessory right colic artery arising from ileo-colic artery

Most cases of an undescended caecum and appendix remain asymptomatic and are incidental findings during autopsy, dissection or laparoscopy [1]. The reduction of the mesentery accompanying an undescended caecum may increase the occurrence of volvulus [13]. Intestinal obstruction can occur due to duodenal compression by Ladd’s bands and may lead to ischaemia and necrosis. These are surgical emergencies accompanied by a sudden onset of abdominal pain, distension, and vomiting [5]. Acute appendicitis may present atypically mimicking acute cholecystitis, renal colic, delaying diagnosis and increasing the chances of complications [4,8,9,11]. The perforation of an inflamed sub-hepatic appendix may be misdiagnosed as liver abscess [9]. The unusual position of the appendicular base or orifice may delay the diagnosis of acute appendicitis as the point of maximum tenderness does not correspond to the McBurney’s point; furthermore, the appendicular base is used as an anatomical landmark in colonoscopy [9]. The raised and atypical position of the ileocaecal junction may interfere with the functioning of the ileocaecal valve [9]. The variant vessels may be inadvertently injured during abdominal surgeries and colonoscopy. We have presented a rare anatomical variation of the right lumbar caecum and appendix with atypical ileocaecal and appendicular orifices and variant blood supply. Understanding such variations is significant for the diagnosis of atypical abdominal pain, clinical decision-making, surgical planning, and for patient safety.

## Limitations

A single case report is insufficient to understand the causative factors of such a developmental anomaly. A rigorously planned study with an adequate sample size of both fetal and adult cases of midgut malrotation is needed for the same objective to be achieved.
